# Prevention of Mastitis

**Published:** 1899-01

**Authors:** 


### PREVENTION OF MASTITIS.

Dr. W. F. B. Wakefield, Oakland, Cal., discusses this question in a paper read before the Alameda County Medical Association (*Occidental Med. Times*, Nov. 1898).

We are told that about 6 per cent. of nursing women are afflicted with mastitis. This is an outrage upon modern obstetrical practice.

One of the most distinguished among British obstetricians, G. Ernest Herman, M. B., F. R. C. P., says: "Sometimes, especially if the nipples are sore, or the child feeble, more milk is produced than the child draws out; the breasts become very full, swollen, knotted, hard, and tender; and this condition may make the patient feverish, and if not relieved may go on to abscess. If the breasts are so full as to be uncomfortable, they should be emptied by a breast-pump or a soda-water bottle. If the child was still-born, or has died, or the mother is not going to suckle her child, this may be repeated as often as necessary, and will soon cease to be required; for if the breasts are deprived of the stimulus of a sucking baby they soon leave off producing milk. It would often be very desirable, in the case of babies too feeble to suck, or nipples too badly shaped for the baby to grasp them, to draw the milk from the breast with a pump and give it to the child with a spoon, but, as a rule, this can not be done, for the breast soon ceases to produce milk when the child is not applied to it. You need not, therefore, prescribe any medicines to drive away the milk. See that the breasts are made comfortable, and the milk will go away if the mother does not suckle."

Again, one of our late American works on obstetrics, Grandin and Jarman, says: "As soon as induration is detected in the gland, nursing should be interdicted, although engorgement of the mamma must be prevented by emptying the breast at regular intervals by the hand. An ice-bag applied over the site of induration may abort it before pus formation."

Such advice in regard to the treatment of "painful fulness of the breasts" is wofully deficient.

The causative factors in the production of mastitis—(1) Bacterial infection through the medium of sore nipples. (2) What Dr. Garrigues, of New York, calls "milk stasis." We pay too little attention to this second etiological factor—"milk stasis." The production of milk is in excess of its consumption, and the breast becomes engorged; and this mechanical engorgement, if not properly handled, soon leads to congestion and inflammation.

*Prevention of Sore Nipples.*—Text-books often recommend that our patient, especially if she be a primipara, put the nipples through a "hardening process," by the use of astringent or spirit lotions, for two or three months prior to confinement. He mentions this simply to condemn it in the most emphatic terms. Nothing is more likely to produce cracked and "sore" nipples than resort to this "hardening" process. Our aim should be, not to harden and stiffen the nipples, but exactly the reverse—to soften and render them more pliable. For five years, he has used, in the treatment of sore or cracked nipples, this formula, recommended by Hirst—

℞—Castor oil.
Subnitrate of bismuth—aa. part equal.
M.—S: Apply freely to the sore nipples.

It has the advantage of being non-toxic, and it is not necessary to wash it off each time the child nurses.

*Treatment of Engorged or Congested Breasts with "Caking."*— The whole treatment consists in firm, evenly directed pressure. Older surgeons, recognizing the necessity for pressure, tried bandaging or strapping the breasts. Neither of these plans gave satisfaction; the bandage slips out of place, and the strapping is very uncomfortable. Garrigues determined to have firm, even pressure applied to the breasts; and Miss Murphy, the Lady Superintendent, invented a breast-binder. It is not necessary to always apply these binders, but one should be in readiness for application whenever the breasts show the slightest sign of hardness, tenderness, or "caking."

Miss Snively, of the Toronto General Hospital, invented a modification of the "Miss Murphy Binder," which can be cut at the bedside, and precludes the necessity of keeping a made-up binder. It has also the advantage of exactly fitting the patient.

To make the bandage, take the bust measurement level with the nipples, cut your muslin one inch longer than this measurement and sixteen inches wide. Fold it twice, and mark off the centre; then measure down four inches on the free edges and make a curved cut to centre; then measure down nine inches on the other side and cut to top, leaving a strip two inches wide. These cuts being made, the binder is ready for application.

In applying the binder, strip the patient to the waist and arrange the binder so that the center of the scallop in the back comes opposite the centre of the back of the neck, bring it around to the front and pin from below upwards, having the patient assist in drawing the breast, as far as possible, upward and inward. In order to have the same pressure on the inside as we have on the outside, a large wad of cotton batting should be placed between the breasts. When the front has been pinned firmly from below upward, pin the shoulder-straps in position to keep the binder from slipping down.

The binder has one drawback, which should ever be kept in view. It diminishes the secretion of milk, and therefore should only be used when the breasts show signs of tenderness and caking. It should only be applied tight enough to relieve the pain; and should be removed as soon as the breasts become soft, and tenderness disappears.—*Virginia Medical Semi-Monthly.*

